# McCabe score as a strong determinant of septic shock-related mortality

**DOI:** 10.1186/1753-6561-5-S6-P74

**Published:** 2011-06-29

**Authors:** F Delodder, Y-A Que, J-P Revelly, P Eggimann, the Staff of the Service of Adult Intensive Care Medicine (SMIA)

**Affiliations:** 1 Intensive Care Medicine, CHUV, Lausanne, Switzerland

## Introduction / objectives

Septic shock is associated with a high mortality. However, we suspected that hospital mortality may be influenced by the predicted outcome of comorbidities and by the origin of the infection

## Methods

We analysed hospital-related mortality of all patients with a septic shock consecutively admitted in our 32-beds mixed university ICU from 2005 to 2008, according to both the origin (community-acquired or nosocomial) and to the underlying McCabe and Johnson score (non fatal, fatal within 5 years, fatal within 6 months). Data are extracted from the clinical information system and combined with a database on case-mix used Following discharge, diagnostic are prospectively validated by the attending physician and further imported in the institution datawarehouse after final crosschecking

## Results

A total of 8979 patients, accounting for 9641 stays were admitted from January 2005 to December 2008. A septic shock was diagnosed in 910 cases, community-acquired and nosocomial in 551 and 358 cases (39.3%), respectively. The McCabe score was nonfatal, fatal within 5 years and fatal within 6 months, in 44.6%, 38.5% and 16.9% of stays, respectively. Overall hospital mortality was 37.0%, 31.1% and 46.0% for all episodes, for community-acquired and nosocomial septic shock, respectively. It was 23.9%, 36.9% and 73.1% for McCabe nonfatal, fatal within 5 years and fatal within 6 months, respectively. Mortality decreased significantly from 73% if nosocomial in patients with an underlying condition scored as potentially fatal within the next 6 months to 20% when community-acquired in a patient with non fatal underlying disease

## Conclusion

The McCabe/Johnson score and the origin (community-acquired or nosocomial) are strong determinant of the outcome of septic shock

## Disclosure of interest

None declared.

